# The Effect of Perioperative Immunonutrition on the Phagocytic Activity of Blood Platelets in Advanced Gastric Cancer Patients

**DOI:** 10.1155/2013/435672

**Published:** 2013-12-01

**Authors:** Zbigniew Kamocki, Joanna Matowicka-Karna, Mariusz Gryko, Konrad Zareba, Boguslaw Kedra, Halina Kemona

**Affiliations:** ^1^2nd Department of General and Gastroenterological Surgery, Medical University of Bialystok, M. Sklodowskiej-Curie 24A Street, 15-276 Bialystok, Poland; ^2^Department of Clinical Laboratory Diagnostics, Medical University of Bialystok, Poland

## Abstract

*Background and Aims*. Perioperative immunonutrition can influence the phagocytic activity of platelets in advanced gastric cancer. *Methods*. 51 patients with stage IV gastric cancer divided into four groups depending on the clinical status and 40 normal donors were analyzed. Patients of groups I and II underwent palliative gastrectomy. Patients of groups III and IV had exploratory laparotomy. Perioperative immunonutrition was administered as follows: group I—TPN, II—oral arginine, peripheral TPN, III—TPN preoperatively, and IV—without nutrition. The phagocytic activity of blood platelets was determined before and after nutritional therapy and was assessed by measuring the fraction of phagocytic thrombocytes (%phag) and the phagocytic index (Ixphag). *Results*. The percentage of phagocytizing platelets and the phagocytic index prior to and after the surgery amounted to the following: group I—1.136–1.237, *P* = NS, and 1.007–1.1, *P* = NS, respectively, II—1.111–1.25, *P* < 0.05, and 1.011–1.083, *P* < 0.05, III—1.112–1.186, *P* = NS, and 0.962–1.042, *P* = NS, and IV—1.085–0.96, *P* = NS, and 1.023–1.04, *P* = NS. *Conclusions*. The phagocytic activity of platelets in patients with advanced gastric cancer is significantly impaired. Perioperative immunonutrition with oral arginine-rich diet can partially improve the phagocytic activity of blood platelets. This trial is registred with Clinicaltrials.gov-NCT01704664.

## 1. Introduction

Surgical treatment of gastric cancer is associated with a high risk of perioperative complications. Morbidity of cancer patients increases in concert with the clinical stage of the malignancy [1]. It is postulated that a reduction in perioperative morbidity and improved quality of life of patients with advanced gastric cancer can be achieved by proper preparation to surgery, among others. One of such methods is the implementation of immunostimulating nutritional therapy during the perioperative period [2–4].

Although the high number and wide distribution within the circulatory system make thrombocytes an important component of immune system, their role in immune response is not fully understood. They are capable of chemotaxis and diapedesis, which enable them to interact with bacteria and viruses [5, 6]. Moreover, they can phagocytize antigen-antibody complexes, collagen, and latex particles. The phagocytic activity is shown both by a single thrombocyte and by the platelets aggregates [7]. The activated thrombocyte changes its discoid shape to an irregular form containing numerous pseudopodia. This is accompanied by the intensification of energetic processes, enhanced protein anabolism inside the platelet [8], as well as the centralization of cellular organelles, and degranulation. Thrombocyte granules contain strong bactericidal compounds such as acidic phosphatase, peroxidase, cationic proteins, and proteolytic enzymes [9].

## 2. Role of Blood Platelets in Neoplastic Disease

Although Billroth postulated that the presence of platelet-composed blood clot is vital for the existence of cancer cells as long as 100 years ago [10], the role of thrombocytes in neoplastic disease is still not fully explained. Studies conducted in 1960s revealed that thrombocytopenia is associated with lower prevalence of metastases [11]. The platelets play a vital role in the formation of metastases as they form aggregates with cancer cells and induce neoangiogenesis via platelet-associated angiogenic factors (VEGF, bFGF, and angiopoietin 1) [12]. Vascular endothelial growth factor (VEGF), which is released from the platelets, stimulates the development of megakaryocytes and thrombocytes and activates the secretion of von Willebrand factor (vWF) from endothelial cells. The latter factor facilitates binding platelets to epithelium via GPIIb-IIIa and GPIa receptors [13]. Moreover, cancer cells release the tissue factor (TF), which also enhances adhesion of activated platelets to epithelium [14]. This transmembrane protein is also involved in cell-to-cell signaling, cellular migration, and development of inflammation. P-selectins, stored in alpha-granules, also play an important role in the process of activation. The activation of platelets is reflected by the expression of P-selectins on the cellular surface. Furthermore, thrombin and ADP activate integrin adhesion molecule GPIIb-IIIa on the platelet surface. This allows the platelets to bind to each other and to the micromolecules of the extracellular matrix, such as fibrinogen, fibronectin, vitronectin, and von Willebrand factor. Activated platelets spread the macromolecules that can be observed as microscopic vesicles located on the cell surface. It was revealed that their serum level is significantly higher in gastric cancer patients than in healthy individuals [15]. Moreover, the concentration of platelet micromolecules was observed to increase together with cancer stage. Platelets protect cancer cells against the cytolytic activity of the immune cells or cytokines released by them, for example, tumor necrosis factor alpha. The endothelial adhesion of platelets and their aggregation occurs at the site of initiated angiogenesis [16]. Activation of clotting cascade is reflected by the dissociation of dissolved fibrinogen into fibrinopeptides and monomers. Insoluble threads of fibrin are formed as a result of the polymerization of monomers, which is catalyzed by platelet factor 4 [17]. The fibers of fibrin that surround cancer cells form a protective barrier, which hinders their destruction by cytotoxic immune cells. Moreover, fibrin facilitates binding of platelets to circulating cancer cells, and subsequently to vascular endothelium. The release of factors stimulating proliferation of cancer cells, including epidermal growth factor (EGF), platelet-derived growth factor (PDGF), and insulin-like growth factor 1 (IGF-1), is seen as another mechanism [18, 19]. Furthermore, the platelets stabilize the binding of cancer cells to the vascular endothelium. The platelets that are bound to cancer cells release VEGF, which increases vascular permeability. Nitric oxide (NO) plays an important role at this stage as it induces the mitogenic properties of VEGF, stimulates the migration of endothelial cells, and loosens vascular endothelial monolayer [20]. Therefore, platelets facilitate extravasation of cancer cells, that is, their migration from the vascular bed to the tissues. Moreover, platelets increase the adhesion of cancer cells to extracellular matrix at the target site of metastasis [21]. Prourokinase plasminogen activator induces the transformation of plasminogen into active plasmin. This in turn degrades the components of basal membrane and extracellular matrix and constitutes strong enzymatic system activating collagenases. Cancer cells can penetrate to tissues distant from primary focus as a result of the activity of these latter enzymes.

In conclusion, platelets play an important role in the development of neoplastic disease; it must be noted, however, that the mechanism of their action is multidirectional. The involvement of platelets in the pathogenesis of neoplastic disease substantiates research on their role in the protective mechanisms of human body. A correct phagocytic activity of blood platelets is an important component of immune system against bacterial infection in postoperative period and perhaps it allows to release ability to phagocytize neoplasmatic cells.

## 3. Objective

The aim of our study was twofold. Firstly, we assessed the efficacy of two methods of perioperative nutritional treatment in stage IV gastric cancer patients subjected to palliative gastrectomy; we analyzed perioperative morbidity and the phagocytic activity of blood platelets in this group. Secondly, we assessed the phagocytic activity of platelets in stage IV gastric cancer patients who received preoperative nutritional treatment.

## 4. Materials and Methods

Amongst 132 patients treated due to gastric cancer during the recent 5 years, 51 individuals (38.3%) were diagnosed with clinical stage IV. This study included 51 patients treated surgically for advanced gastric cancer (the IV stage) and the 40 healthy volunteers, control group. The stage of cancer was graded according to TNM classification approved by the International Union Against Cancer (UICC). The patients were classified into groups depending on their clinical status. Group I comprised patients in whom cancer was associated with severe obstruction disallowing oral nutrition. Group II included individuals who had good tolerance of nutrition via the natural route. In group II, exclusion criterion constituted gastric cancer associated with severe gastrointestinal obstruction.

Group III included patients who were originally qualified to palliative gastrectomy, but the gastric resection was aborted intraoperatively due to excessively advanced peritoneal spread of cancer, for example, shortening of the mesentery by neoplastic implants, preventing the restoration of the continuity of the gastrointestinal tract after the resection. Patients in whom bleeding from gastric tumor precluded preoperative nutritional treatment underwent emergency surgery. In this group, it was impossible to carry out palliative gastrectomy due to advanced neoplastic spread. Following surgical treatment, all patients were qualified to chemotherapy and radiotherapy.

Total gastroomentectomy was performed in patients of groups I and II. The continuity of gastrointestinal tract was restored with Roux-en-Y esophagojejunostomy. Patients of groups III and IV underwent only laparotomy due to severe simultaneous peritoneal spread of cancer.

Immunonutritional support was used in patients of groups I, II, and III in perioperative time. Groups I and II patients were treated nutritionally both prior to and after the surgery. Patients from group III were administered nutritional therapy solely during the preoperative period.

The patients of group IV were not treated with artificial nutrition.

## 5. Perioperative Nutrition

Nutritional therapy of group I was based on intravenous preparations. Two-chamber bags with 480 kcal energetic value and 5.7 g of N in standard amino acids were administered preoperatively. A solution of glutamine (Dipeptiven, 100 mL) and *ω*3-fatty acids (Omegaven, 100 mL) was added to the bags. The duration of preoperative preparatory phase ranged between 5 and 10 days (8 days on average). Enteral nutrition with elementary commercially available diet (Peptisorb) was begun 20 hours after surgery; it was started at an 8 mL/h flow rate and increased gradually, with the volume doubled every 24 hours, up to 100 mL/h. The enteral nutrition was continued for six days. During the initial five days after surgery, the patients were additionally supplemented parenterally via peripheral veins; similar to the preoperative period, the content of two-chamber bag for peripheral access enriched with glutamine and *ω*3-fatty acids was administered for five days.

Preoperatively, group II patients were given commercially available oral diet enriched with arginine (Cubitan, 1 package 3 times per day). Additionally, they were administered commercially available two-chamber bag with 480 kcal energetic value and 5.7 g of N in standard amino acids via peripheral access. The duration of preoperative preparatory phase ranged between 5 and 10 days (8 days on average). Enteral nutrition with commercially available arginine-containing diet (Cubison) was started 20 hours after surgery at an 8 mL/h flow rate; the rate was increased gradually, with the volume doubled every 24 hours, up to 100 mL/h and continued for six days. Simultaneously, commercially available two-chamber bags for peripheral access with composition identical to that used preoperatively were administered via peripheral veins for five days.

Nutritional therapy of group III was used only before the operation based on intravenous preparations. As in group I, two-chamber bags with 480 kcal energetic value and 5.7 g of N in standard amino acids were administered preoperatively. A solution of glutamine (Dipeptiven, 100 mL) and *ω*3-fatty acids (Omegaven, 100 mL) was added to the bags. The duration of preoperative preparatory phase ranged between 5 and 10 days (8 days on average).

Body mass index, weight loss percentage, total protein level, albumin, cholesterol, and triglicerid level were calculated for each patient prior to and after the operation. Platelet count and phagocytic activity of thrombocytes were examined twice in each patient. Blood samples for laboratory tests were drawn prior to surgery and nutritional therapy and 12 days after the surgery. Thrombocyte count was determined using Advia 120 haematological analyser. Phagocytic activity of blood platelets was determined against *Staphylococcus aureus* ATCC 6538P bacterial strain. It was expressed as the fraction of phagocytizing platelets and the phagocytic index.

## 6. Assessment of Platelet Phagocytic Activity

About 1 mL of ACD (a mixture of citric acid and glucose) was added to 5 mL of venous blood collected for heparin at a concentration of 50 IU/mL blood. ACD prevents platelet aggregation by inhibiting thromboxane synthesis by platelets and reduces plasma pH to 6.5 which decreases the physiological ability of platelets to agglutinate. Blood platelets were isolated by double centrifugation taking advantage of the differences in the specific weight of the respective morphotic elements of blood. Whole blood was centrifuged at 250 g for 12 minutes, plasma was withdrawn to another test-tube, and then centrifugation was repeated at 1500 g for 20 minutes. Some plasma (2 mL) was left over the platelets sediment, and the sedimented platelets were suspended in it by gentle stirring with a plastic pipette. This platelets-rich plasma was supplemented with 0.1 mL ACD to prevent platelets aggregation. In this plasma erythrocytes and leukocytes did not exceed 1/1000 platelets.

## 7. Bacteria Used for the Phagocytic Activity of Blood Platelets

*Staphylococcus aureus* ATCC 6538P strain was used for the study. The bacteria were cultured on an agar slant for 20 minutes, rinsed off with 10 mL of phosphate buffer (PBS), and centrifuged at 1500 g for 20 minutes. The centrifuged bacterial sediment was rinsed with PBS three times and centrifuged again. The number of bacteria in the suspension was determined spectrophotometrically by measuring optical density of the suspension. The suspension containing 120 × 10^6^ bacteria was used to examine the platelet phagocytic activity, corresponding to an optical density of T-63 at a wavelength of 540 nm. The bacteria were grown on the agar medium and counted. The suspension was diluted with PBS to obtain the platelets/bacterium ratio of 1 : 1.

## 8. Determination of the Percentage of Phagocytizing Platelets and the Phagocytic Index

Bacterial suspension and platelet-rich plasma were incubated at 37°C for 6 minutes and then mixed at 75 g. After incubation smears were made and stained with the Pappenheim method for 1 h using Giemsa reagent. The stained preparations were evaluated in a light microscope at ×1400 magnification. The percentage of phagocytizing platelets was described as the percentage of phagocytizing platelets per 1000 other cells in the preparation. The phagocytic index was determined using 100 phagocytizing platelets. The index was calculated as the mean number of phagocytized bacteria per single platelet according to the following formula: phagocytic index = number of phagocytized bacteria/number of phagocytizing platelets.

## 9. Statistical Analysis

A given data set has a mean and a standard deviation. The results were subjected to statistical analysis with the Mann-Whitney test, and the statistical significance was defined at *P* < 0.05.

## 10. Results

The groups consisted of the following number of patients: group I—19, group II—10, III—11, and group IV—11 (Table 1).

The patients from study groups were older than the control group, but there were no significant differences in age between examined groups. No significant differences in body mass index in study groups and control group were observed. A loss of more than 10% of body weight was documented in 17 patients, corresponding to 89% of group I. All patients (100%) from group II experienced a loss of more than 10% of body weight. In group III more than 10% reduction in body weight was documented in 10 patients, corresponding to 90.9%.

All patients (100%) from group IV experienced a loss of more than 10% of body weight.

There were no differences in levels of total proteins, serum albumins, cholesterols, and triglycerides between each group (Table 2). Only in group IV the level of total protein was significantly lower.

In group I patients total protein levels and serum albumin levels significantly decreased after the therapy. The cholesterol levels and triglycerides decreased. This therapy-related decrease in cholesterol levels and triglycerides did not prove to be significant on statistical analysis (*P* = NS). In group II patients total protein levels and serum albumin levels decreased after the therapy also, but nonsignificantly on statistical analysis. The therapy-related decrease in cholesterol levels and therapy-related increase in triglycerides levels did not prove to be significant on statistical analysis.

In groups III and IV patients therapy-related decrease in total protein levels, serum albumin levels, cholesterol, and triglycerides was statistically insignificant (*P* = NS).

16 total gastrectomies and 3 subtotal gastrectomies were performed in patients of group I. In this group, 5 (26,3%) patients had early complications. In group II, 9 patients had total gastrectomy and one patient underwent proximal gastrectomy. Complications occurred in 2 (20%) of 10 patients. The complications are outlined in Table 3.

## 11. Blood Platelets and Their Phagocytic Activity

### 11.1. Group I

Baseline thrombocyte count in group I patients ranged between 205,000.0 and 532,000.0 per mm^3^ (mean 311,714.0 per mm^3^). It was significantly higher compared to the normal donors. In control group, the average number of blood platelets was 229,150.0/*μ*L (124,000.0–384,000.0/*μ*L). Significant increase in thrombocyte count to 267,000.0–1,113,000.0 per mm^3^ (mean 645,900.0 per mm^3^) was observed after the therapy (*P* < 0.05). Pre- and posttherapy thrombocyte counts are presented in Table 4.

Prior to treatment, the fraction of phagocytizing platelets in group I patients ranged between 1.0% and 1.3% (mean 1.136%). It was significantly lower compared to the normal donors 2.259% (1.2–3.6%). The fraction of phagocytizing platelets increased after therapy; this change, however, did not prove to be statistically significant (*P* > 0.05). The posttreatment fraction of phagocytizing thrombocytes ranged between 1.1% and 1.4% (mean 1.237%). Pre- and posttherapy fraction of thrombocytizing thrombocytes is presented in Table 5.

The pretreatment phagocytic index of thrombocytes in group I patients ranged between 0.8 and 1.2 (mean 1.007). It was significantly lower compared to the normal donors 1.828 (1.1–2.57). Slightly higher values of this parameter were observed after completing nutritional therapy; the posttherapy phagocytic index ranged between 1.0 and 1.2 (mean 1.1). Pre- and posttherapy phagocytic index of thrombocytes is presented in Table 6.

### 11.2. Group II

Baseline blood platelets count in group II patients ranged between 276,000.0 and 393,000.0 per mm^3^ (mean 335,889.0 per mm^3^). Significant increase in thrombocyte count to 149,000.0–1,038,000.0 per mm^3^ (mean 769,333.0 per mm^3^) was observed after the therapy (*P* < 0.05). Pre- and posttherapy thrombocyte counts are presented in Table 4.

Prior to treatment, the fraction of phagocytizing platelets in group II patients ranged between 1.0% and 1.2% (mean 1.111%). It was significantly lower compared to the normal donors. The fraction of phagocytizing platelets increased statistically significant after therapy, (*P* < 0.05). The post-treatment fraction of phagocytizing thrombocytes ranged between 1.0% and 1.4% (mean 1.257%). It was still significantly lower compared to the normal donors (Table 5).

The pretreatment phagocytic index of thrombocytes in group II patients ranged between 0.9 and 1.1 (mean 1.011). It was significantly lower comparing o the normal donors. The index increased statistically significant after therapy, (*P* < 0.05). The posttherapy phagocytic index ranged between 1.0 and 1.1 (mean 1.083). It was still significantly lower compared to the normal donors. Pre- and posttreatment values of phagocytic index in group II patients are illustrated in Table 6.

### 11.3. Group III

Baseline thrombocyte count in group III patients ranged between 160,000.0 and 597,000.0 per mm^3^ (mean 283,250.0 per mm^3^). It was significantly higher compared to the normal donors. The blood platelets count did not increased significantly after the therapy, (*P* > 0.05). It ranged between 202,000.0 and 558,000.0 per mm^3^ (mean 354,285.0 per mm^3^). Pre- and posttherapy thrombocyte counts are presented in Table 4.

Prior to treatment, the fraction of phagocytizing platelets in group III patients ranged between 1.0% and 1.2% (mean 1.112%). It was significantly lower compared to the normal donors. Slightly higher values of this parameter were observed after completing nutritional therapy (*P* > 0.05). The post-treatment fraction of phagocytizing thrombocytes ranged between 1.1% and 1.3% (mean 1.186%), Table 5.

The pre-treatment phagocytic index of thrombocytes in group III patients ranged between 0.8 and 1.1 (mean 0.962). It was significantly lower compared to the normal donors. Therapy-related increase in phagocytic index did not prove to be significant on statistical analysis (*P* > 0.05). The posttherapy phagocytic index ranged between 0.9 and 1.2 (mean 1.043). Pre- and post-treatment values of phagocytic index in group III patients are presented in Table 6.

### 11.4. Group IV

Baseline blood platelets count in group IV patients ranged between 165,000.0 and 493,000.0 per mm^3^ (mean 259,909.0 per mm^3^). No significant differences in the number of platelets in patients from group IV and normal donors were observed. The number of platelets did not increase significantly after the therapy (*P* > 0.05). The thrombocyte count ranged between 42,000.0 and 636,000.0 per mm^3^ (mean 287,500.0 per mm^3^). Pre- and posttherapy thrombocyte counts are presented in Table 4.

Prior to treatment, the fraction of phagocytizing platelets in group IV patients ranged between 0.8% and 1.3% (mean 1.085%). It was significantly lower compared to the normal donors. The post-treatment fraction of phagocytizing thrombocytes ranged between 0.6% and 1.1% (mean 0.96%), Table 5.

The pre-treatment phagocytic index of thrombocytes in group IV patients ranged between 0.9 and 1.2 (mean 1.023). It was significantly lower compared to the normal donors. The posttherapy phagocytic index did not prove to be significant on statistical analysis *P* = NS. It ranged between 0.9 and 1.3 (mean 1.023) Table 6.

**Figure 1:**
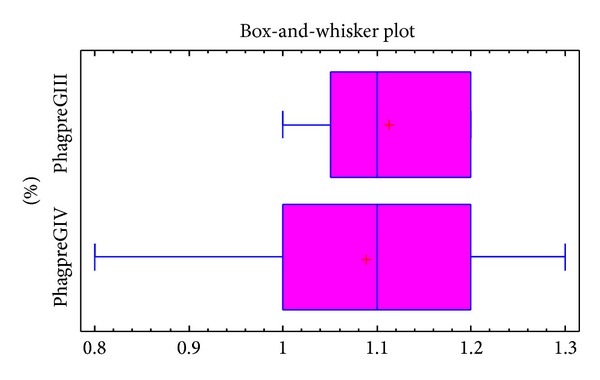
Values of phagocytizing platelets in patients of group III (%phagpreGIII) and group IV (%phagpreGIV) patients determined before the treatment.

There was no difference in the preoperative percentage of phagocyizting blood platelet between patients from groups III and group IV, *P* = NS, Figure 1.

**Figure 2:**
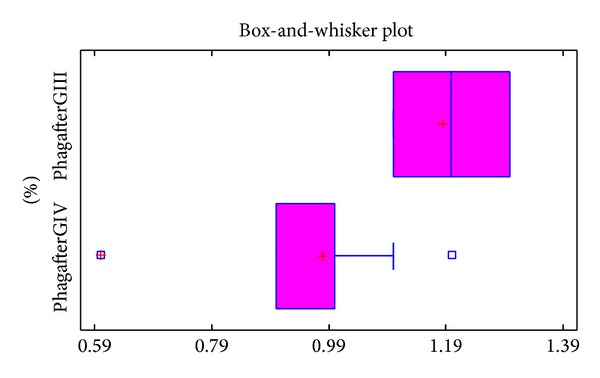
Values of phagocytizing platelets in patients of group III (%phagafterGIII) and group IV (%phagafterGIV) patients determined following the laparotomy.

After laparotomy the percentage of blood platelets in patients of group IV was significantly lower compared to group III patients. Post-treatment values of phagocytizing platelets in groups III and IV patients are illustrated in Figure 2.

There was no difference both in the preoperative and post-treatment values of phagocytic index between patients from group III and group IV, *P* = NS.

## 12. Discussion 

Platelets can have a direct impact on tumour cell behavior. The molecular mechanisms induced by platelet-tumour cell interactions influence increased metastatic capacity of tumour cells [22]. But little is known about the phagocytic activity of blood platelets in gastric cancer patients.

### 12.1. Task I

The efficacy of perioperative nutritional treatment in patients subjected to palliative gastrectomy was analyzed on the basis of results obtained in groups I and II. Patients from both groups showed a similar degree of malnutrition. No perioperative mortality was documented in either group. The rates of severe postoperative complications were similar and equal to 26.3% and 20% in groups I and II, respectively. A significant decrease in total protein and albumin concentration was documented postoperatively in group I, whereas the decrease in these parameters in group II patients proved to be insignificant. Observed changes in cholesterol and triglyceride concentration were not significant. It can be assumed that the efficacy of perioperative immunostimulant nutritional therapy based on oral arginine solutions is similar as in the case of immunostimulant parenteral nutrition. Braga revealed that oral immunostimulant diet administered for 7 days prior to the surgery and continued postoperatively in parenteral form for another week significantly reduces postoperative infectious morbidity and reduces length of hospital stay [23]. The phagocytic activity of platelets in both studied groups was significantly lower than that in healthy individuals. The immunostimulant nutrition did not modulate the phagocytic activity of platelets in group I. In contrast, group II showed a significant increase in the phagocytic activity, regarding both the percentage of phagocytic platelets and the phagocytic index. These findings substantiate the use of immunostimulant enteral nutrition in patients with advanced gastric cancer who were qualified to palliative gastrectomy.

### 12.2. Task II

We analyzed the results of individuals from groups III and IV to verify the influence of preoperative immunostimulant enteral nutrition of the phagocytic activity of platelets in patients with advanced gastric cancer. The stage of cancer was similar in both groups. Moreover, these groups did not differ significantly in terms of laboratory indices of nutritional status, aside from decreased total protein concentration in group IV. Explorative laparotomy was the only procedure performed in both groups; the only difference in treatment pertained to the application of preoperative immunostimulant nutritional therapy in Group III. The phagocytic activity of platelets in both groups was significantly lower than that in healthy individuals; however, there were no significant intergroup differences in this parameter. An insignificant increase in the percentage of phagocytic platelets and phagocytic index was documented after laparotomy in group III. In contrast, the postoperative levels of these two parameters slightly decreased in group IV, but these changes also proved to be insignificant compared to respective baseline values. The postoperative percentage of phagocytic platelets and the postoperative phagocytic index in group III were significantly higher than those in group IV. These findings attest to the positive effect of preoperative immunostimulant nutritional therapy in patients with nonresectable spread of gastric cancer as this modality allows for retaining baseline phagocytic activity of blood platelets.

Our study shows that the phagocytic activity of thrombocytes in advanced gastric cancer patients is lower than that in healthy individuals. Both the fraction of phagocytizing platelets and the phagocytic index were decreased in all patients. This study of advanced gastric cancer patients revealed a relationship between the postgastrectomy increase in the phagocytic activity of thrombocytes and the type of nutritional therapy administered perioperatively. The phagocytic activity of thrombocytes increased only partially in the group of patients in whom immunonutritional therapy was implemented only intravenously in perioperative period. A very interesting finding arising from the present study is the significant improvement in the phagocytic activity of thrombocytes in patients who received preoperative oral arginine solutions. Significant increase in the fraction of phagocytizing thrombocytes was observed in this group along with the higher number of bacteria phagocytized by a single platelet. This implies that perioperative approach in gastric cancer patients with retained gastrointestinal passage can be relatively easy and associated with marked reduction in treatment costs. The results of this study could have important clinical significance. For instance, improvement in phagocytic and bactericidal activity of blood platelets can be reflected by the lower number of infectious complications associated with gastrectomy. The results in group IV patients confirm an influence immunonutrition on the phagocytic activity of blood platelets in advanced gastric patients. Therapy without immunonutrition was reflected by a significant decrease in both the fraction of phagocytizing platelets and the phagocytic index after laparotomy. Our previous studies of gastric cancer patients revealed a significant impairment in bactericidal activity of thrombocytes. Furthermore, platelets of patients with nonresectable gastric cancer were not able to destroy bacteria [24]. The results of present study confirm our preliminary findings in advanced gastric cancer. Both the development of gastric cancer and its combined therapy are associated with immunosuppression of the patient; therefore, the perioperative nutritional therapy should modulate immune response.

## 13. Conclusions

1. The phagocytic activity of blood platelets in patients with advanced gastric cancer is significantly impaired.
2. Perioperative immunonutrition can partially improve the phagocytic activity of blood platelets.
3. Oral solutions of arginine, administered preoperatively along with adjunct nutritional therapy in advanced gastric cancer patients with retained gastrointestinal passage, significantly increase the fraction of phagocytizing platelets and improve the phagocytic index of thrombocytes.

## Figures and Tables

**Table 1:** Gender, age, and body mass index (BMI) in all groups of patients, and in control group.

	Gender Female/male (all)	Age (years) Min–Max (mean)	BMI (kg/m^2^) Min–Max (mean)
Group I	8/11 (19)	37–80 (63.4)	18–27 (22.8)
Group II	1/9 (10)	42–67 (54.8)	20–27 (23.0)
Group III	3/8 (11)	46–77 (59.6)	16–28 (22.1)
Group IV	6/5 (11)	31–82 (59.1)	16–29 (23.5)
Control	22/18 (40)	18–46 (28.6)*	21–26 (23.6)

*P < 0.05.

**Table 2:** The mean value of total protein, serum albumin, cholesterol, and triglyceride in all groups of patients prior to the treatment and after the treatment.

	Protein (g/dL)		Albumin (g/L)		Cholesterol (mg/dL)		Triglyceride (mg/dL)	
	Before	After	Before	After	Before	After	Before	After
Group I	6,19	5,62	34,5	26,9	170,7	147,2	91,8	114,0
Group II	6,26	5,74	33,2	28,6	170,1	126,8	104,5	146,2
Group III	5,93	5,68	31,1	27,5	129,0	140,0	99,9	141,5
Group IV	5,64*	5,7	29,9	28,0	155,0	152,0	92,0	91,5

*P < 0.05.

**Table 3:** Complications after total gastrectomy.

	Oesophagojejunal leak	Duodenal fistula	Pancreatic fistula	Subphrenic abscess
Group I	1	1	1	2
Group II		1		1

**Table 4:** Mean thrombocyte counts and standard deviation of all groups patients prior to and following the completion of nutritional therapy compared with the control group.

	Before the treatment (/mm^3^)	After the treatment (/mm^3^)	Statistical analysis Mann-Whitney test (before/after treatment)	Control group	Statistical analysis Mann-Whitney test (control/before the treatment)	Statistical analysis Mann-Whitney test (control/after the treatment)
Group I	311,714.0 ± 103,470.0	645,000.0 ± 273,777.0	*P* < 0.005	229,150.0 ± 52,505,0	*P* < 0.005	*P* < 0.001
Group II	335,889.0 ± 39,011.0	769,333.0 ± 334,336.0	*P* < 0.05	229,150.0 ± 52,505,0	*P* < 0.001	*P* < 0.01
Group III	283,250.0 ± 152,092.0	354,286.0 ± 108,657.0	*P* = NS	229,150.0 ± 52,505,0	*P* = NS	*P* < 0.005
Group IV	259,909.0 ± 90,246.8	287,500.0 ± 166,695.0	*P* = NS	229,150.0 ± 52,505,0	*P* = NS	*P* = NS

**Table 5:** Fraction of phagocytizing thrombocytes and standard deviation of all groups patients prior to and following the completion of nutritional therapy compared with the control group.

	Before the treatment (%)	After the treatment (%)	Statistical analysis Mann-Whitney test (before/after treatment)	Control group	Statistical analysis Mann-Whitney test (control/before the treatment)	Statistical analysis Mann-Whitney test (control/after the treatment)
Group I	1.136 ± 0.09	1.237 ± 0.13	*P* = NS	2.259 ± 0.57	*P* < 0.001	*P* < 0.001
Group II	1.111 ± 0.08	1.25± 0.1	*P* < 0.05	2.259 ± 0.57	*P* < 0.001	*P* < 0.001
Group III	1.112 ± 0.08	1.18 ± 0.09	*P* = NS	2.259 ± 0.57	*P* < 0.001	*P* < 0.001
Group IV	1.084 ± 0.15	0.96 ± 0.16	*P* = NS	2.259 ± 0.57	*P* < 0.001	*P* < 0.001

**Table 6:** Phagocytic index of thrombocytes and standard deviation of all groups patients prior to and following the completion of nutritional therapy compared with the control group.

	Before the treatment	After the treatment	Statistical analysis Mann-Whitney test (before/after treatment)	Control group	Statistical analysis Mann-Whitney test (control/before treatment)	Statistical analysis Mann-Whitney test (control/after treatment)
Group I	1.007 ± 0.11	1.1 ± 0.09	*P* = NS	1.828 ± 0.37	*P* < 0.001	*P* < 0.001
Group II	1.011 ± 0.06	1.083 ± 0.04	*P* < 0.05	1.828 ± 0.37	*P* < 0.001	*P* < 0.001
Group III	0.962 ± 0.11	1.043 ± 0.1	*P* = NS	1.828 ± 0.37	*P* < 0.001	*P* < 0.001
Group IV	1.023 ± 0.09	1.04 ± 0.14	*P* = NS	1.828 ± 0.37	*P* < 0.001	*P* < 0.001
